# Magnetic Resonance Sensors

**DOI:** 10.3390/s141121722

**Published:** 2014-11-17

**Authors:** Robert H. Morris, Michael I. Newton

**Affiliations:** School of Science and Technology, Nottingham Trent University, Clifton Lane, Nottingham, NG11 8NS, UK; E-Mail: michael.newton@ntu.ac.uk

Magnetic Resonance finds countless applications, from spectroscopy to imaging, routinely in almost all research and medical institutions across the globe. It is also becoming more frequently used for specific applications in which the whole instrument and system is designed for a dedicated application. With beginnings in borehole logging for the petro-chemical industry Magnetic Resonance sensors have been applied to fields as varied as online process monitoring for food manufacture and medical point of care diagnostics. This great diversity is seeing exciting developments in magnetic resonance sensing technology published in application specific journals where they are often not seen by the wider sensor community. It is clear that there is enormous interest in magnetic resonance sensors which represents a significant growth area. The aim of this special edition of *Sensors* was to address the wide distribution of relevant articles by providing a forum to disseminate cutting edge research in this field in a single open source publication.

## Summary of the Special Issue

Of particular importance to this community is the use of small, low field instruments, the development of which has been made possible by the steady progress in permanent magnet technology. In our first two contributions we have examples of *in-situ* monitoring using such sensors: Firstly for assessing compressive strength and pore size distribution in concrete [1] where the results indicated that the sensor is capable of detecting changes in water content in fresh cement pastes and porosity refinement caused by cement hydration in hardened materials, even if they are prepared with a low water/cement ratio; Secondly to assess the degradation of hydraulic fluid in power station turbines [2]. Here, a three-magnet unilateral NMR sensor array was used in two different power stations for assessing aging of the turbine oils used. Their results showed that components of the longitudinal and transverse relaxation times shortened as the fluids aged providing a useful tool for monitoring hydraulic fluids in turbines. These two articles highlight the trend in magnetic resonance sensors towards offering sensor applications which are distinct from imaging or spectroscopy but which provide valuable information to the operator. Magnetic resonance also offers the opportunity to non-destructively monitor materials in sealed containers such as food and drink products. The next two articles report on important aspects of this branch of sensor technology: The detection of adulteration of olive oil is reported in [3] using a unilateral NMR MOUSE and applying a 2-dimensional inverse Laplace transformation. The adulteration of extra virgin olive oil with different percentages of sunflower oil or red palm oil are shown to be quantitatively different using the transverse relaxation and self-diffusion coefficients of the bulk sample. In [4] it was found that spoilage in tomato paste test samples leads to longer spin lattice relaxation times and that it is possible to use a unilateral instrument through a standard non-ferrous, metal-lined bulk storage container to collect these signals non-invasively. Crucial for the widening the applications of magnetic resonance sensors is the availability of low cost, low power console electronics which is discussed in [5] where the authors demonstrate a proof-of-concept MR console system which is fully digital and constructed using off the shelf equipment wherever possible. The authors present such a system based on a Direct Digital Synthesizer (DDS) used to produce the pulses, a Software Defined Radio (SDR) do digitally collect and process the resultant NMR signal and a Digital Signal Processor (DSP) as the central processing unit.

A second aspect of the papers presented in this special issue is the combining of traditional MRI imaging with other sensors. A review of optical fiber sensors that are MRI compatible is presented in [6], focusing on the sensors employed for measuring physical parameters in medicine (*i.e.*, temperature, force, torque, strain, and position) including working principles and their relevant advantages and disadvantages. This may, in the future see interesting combinations with other technologies such as with gated MRI modalities like the fetal electrocardiogram triggered MRI reported in [7]. Here the authors have successfully imaged a stationary slice through a fetal heart despite the significantly higher heart rate in comparison to adult patients and the indirect connection of ECG leads. Finally in this section, in [8] an applied example of the fiber optic sensors discussed in [6] is demonstrated where the authors used a fiber-optic Fabry-Perot interferometer pressure transducer to record two transient characteristics of the consolidation of articular cartilage: the change over time of strain and the hydrostatic excess pore pressure (HEPP).

In traditional imaging, it is sometimes the case that there is no intrinsic contrast to the pathology of interest. To address this MRI contrast agents that consist of Gd(III) or other Lanthanide chelates are often used to enhance the image contrast of anatomical features. The difficulty with their use is that *ex vivo* tissue concentrations are often required. In [9] the authors present a method which uses changes in magnetic susceptibility to determine the concentration of a range of Lanthanide chelates, using NMR spectroscopy. Although presented using a high field spectrometer, this could easily be performed in the clinical setting using a bench top spectrometer system such as the Magritek Spinsolve [10] improving the efficiency at point of care. An application of such contrast agents is considered in [11] which reviews advances in our understanding of stroke pathophysiology with imaging. This article investigates the ability to image tissue viability post-stroke using MRI with and without paramagnetic contrast agents. An alternative approach to the use of a contrast agent is the application of contrast enhancement. In [12] Spin hyperpolarization is reviewed for its use in the analysis of biological assays to detect parameters within the cellular structure such as intracellular reaction progression, drug efficacy, pathway kinetics, probe uptake and export, oncogenic signaling, redox state, ion concentrations, reactive oxygen species or pH.

Our final two papers present methods to improve the way in which imaging is conducted. When setting up an MRI scan, there are numerous choices open to the operator for parameters which impact the final result. In many cases, standard settings are used which are not optimal in terms of patient scan time, image quality or contrast. This is in part due to a lack of optimization schemes which can be used to determine the optimum values for various parameters. This has been addressed in [13] where the authors present a number of recipes are presented for optimum settings in spin echo imaging modalities at 1.5 T and 3 T. Another issue which is increasingly problematic in the clinical setting is the calibration of parallel MRI which is a technique where multiple receive coils are used to improve the signal to noise ratio. Traditionally, parallel imaging requires the use of parameter estimation or calibration prior to scanning. In [14] the authors present a new technique which requires no such calibration prior to use and yet which offers better reconstruction than alternative techniques currently available. Where implemented this will result in improved clinical images and reduced patient scan time.

To conclude, we have compiled a special issue in which we have aimed to address the disparity of publications related to magnetic resonance sensors, both from the point of view of customized probes and the sensors used with imaging or spectroscopy techniques. We would like to thank all the authors who submitted manuscripts to this Special Issue of *Sensors* for preparing such interesting and varied work and the reviewers for their careful consideration and constructive criticism during the rigorous review process.
